# Clinical outcomes and visual prognostic factors in congenital aniridia

**DOI:** 10.1186/s12886-022-02460-5

**Published:** 2022-05-25

**Authors:** Adam Jacobson, Shahzad I. Mian, Brenda L. Bohnsack

**Affiliations:** 1 Department of Ophthalmology and Visual Sciences, University of Michigan, 1000 Wall Street, Ann Arbor, MI 48105 USA; 2 Division of Ophthalmology, Ann & Robert H. Lurie Children’s Hospital of Chicago, 225 E. Chicago Ave, Chicago, IL 60611 USA; 3 Department of Ophthalmology, Northwestern University Feinberg School of Medicine, 645 N. Michigan Ave, Chicago, IL 60611 USA

**Keywords:** Aniridia, Glaucoma, Glaucoma drainage device, Keratopathy, Keratoplasty, Cataract

## Abstract

**Background:**

Evaluate outcomes and identify prognostic factors in congenital aniridia.

**Methods:**

Retrospective interventional case series of patients with congenital aniridia treated between 2012–2020. Ocular examination and surgical details were collected. Surgical failure was defined as disease progression or need for additional surgery for same/related indication. Kaplan–Meier survival curves, Wilcoxon test, and univariate and multivariate linear regression analyses were performed.

**Results:**

Ninety-four patients with congenital aniridia presented at median 19.0 years. Two-thirds of patients underwent ≥ 1intraocular surgery, with average of 1.7 ± 2.3 surgeries/eye. At final follow-up (median 4.0 years), 45% of eyes had undergone lensectomy. Aphakic eyes showed worse visual acuity (VA) than phakic or pseudophakic eyes. Glaucoma affected 52% of eyes, of which half required IOP-lowering surgery. Glaucoma drainage devices showed the highest success rate (71%) at 14.2 ± 15.4 years of follow-up. Keratopathy affected 65% of eyes and one-third underwent corneal surgery. Keratoprosthesis had the longest survival rates at 10-years (64% with 95% CI [32,84]). LogMAR VA at presentation and final follow-up were not statistically different. Half of patients were legally blind at final follow-up. Final VA was associated with presenting VA, glaucoma diagnosis, and cataract or keratopathy at presentation. Penetrating keratoplasty and keratoprosthesis implantation correlated with worse BCVA.

**Conclusions:**

Most aniridic patients in this large US-based cohort underwent at least 1 intraocular surgery. Cataract, glaucoma, and keratopathy were associated with worse VA and are important prognostic factors to consider when managing congenital aniridia.

**Supplementary Information:**

The online version contains supplementary material available at 10.1186/s12886-022-02460-5.

## Background

Despite being named for iris hypoplasia, congenital aniridia affects almost all eye structures. Optic nerve and foveal hypoplasia cause early visual impairment, while cataracts, refractory glaucoma and keratopathy result in progressive vision loss [1–4]. Congenital aniridia is typically due to autosomal dominant mutations in *PAX6* [3, 5–7]. Approximately 2/3 of cases are familial while 1/3 are sporadic. A small proportion of the sporadic cases have WAGR syndrome (OMIM194072), a 11p13 contiguous gene deletion syndrome involving *PAX6* and *WT1* genes [7, 8].

Aniridia reports have focused on outcomes of one aspect, such as cataracts, glaucoma, or keratopathy [9–16]. Few studies, most European or Asian based, have examined the epidemiology and overall clinical outcomes [17–20]. In the current study, we present clinical courses and visual outcomes of one of the largest US-based cohort of aniridic patients. We aimed to determine prognostic factors that influence vision in order to help guide the care of individuals with aniridia.

## Methods

A retrospective review identified patients with congenital aniridia at the University of Michigan from August 2012-April 2020. This retrospective study was approved by the Institutional Review Board at the University of Michigan as exempt such that obtaining informed consent from patients was waived. Collected data included age, gender, family history, ocular and systemic diagnoses, genetic mutation, and intraocular surgeries. Examination findings including initial and final best corrected visual acuity (BCVA), intraocular pressure (IOP), refractive error, slit lamp, and fundus exam findings were recorded.

Optotype BCVA was converted to LogMAR format. Non-optotype vision recorded as counting fingers, hand motion, light perception, and no light perception were approximated as 1.9, 2.3, 2.7, and 3.0 LogMAR, respectively, based on previously published guidelines [21–23]. Vision in non-verbal patients that was recorded as “central steady and maintained”, “reacts to light”, “prefers/holds”, was not incorporated in statistical testing. A change in BCVA between presentation and final follow-up was defined as a difference of ≥ 0.1 LogMAR.

Glaucoma in children (< 18 years of age) as defined by the World Glaucoma Association was at least 2 repeated intraocular pressure measurements greater than 21 mmHg and accompanying signs of corneal edema, Haabs striae, optic nerve cupping, or buphthalmos [24]. Glaucoma in adult patients was defined as optic nerve damage (cupping) with associated optical coherence tomography and visual field findings. Failure of glaucoma surgery was defined as IOP less than 5 mmHg or greater than 21 mmHg (with or without glaucoma medications) for two consecutive visits, the need for additional IOP-related surgery, or a visually-devastating complication (e.g. suprachoroidal hemorrhage, retinal detachment, endophthalmitis).

Keratopathy was defined as central or peripheral neovascularization with stromal haze, scarring, or thinning, regardless of visual significance. Symptoms of dry eyes alone or isolated superficial punctate keratopathy without additional anatomical changes were not considered a positive history of aniridic keratopathy [10, 25, 26]. Failure of corneal surgeries, including corneal transplantation or limbal stem cell transplantation, was defined by graft failure or the need for additional corneal surgery.

Statistical analyses were performed with SPSS (IBM Corp, Armonk, NY). Univariate and multivariate linear regression was used to test for statistical significance of covariates with final LogMAR BCVA. Surgical failure was defined as further progression of the disease, the need for repeat surgery, or additional surgery for the same or related indication. A *p*-value of < 0.05 was considered statistically significant.

## Results

Ninety-four patients with congenital aniridia were identified, of which 46 (49%) were male. Median age at presentation was 19.0 years (mean 23.7 ± 21.6 years, range 0–84). Self-identified racial ethnicity was Caucasian (87%), Black (9%), Asian (3%), and Hispanic (1%). Fifty-one patients (54%) had positive family history (familial) while 28 (30%) were documented to have no family history (sporadic). The remaining 15 patients (16%) did not have family history documented in the medical record. There were no differences in sex (*p* = 0.85), race (*p* = 0.85) or follow up length (*p* = 0.235) amongst aniridic patients classified as familial, sporadic, and unknown family history.

**Fig. 1:**
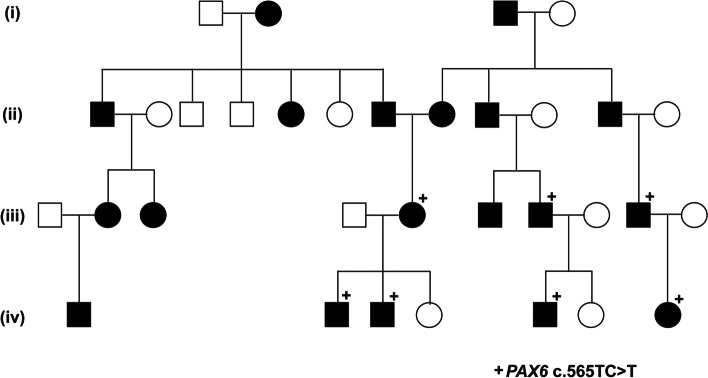
Pedigree of 4-Generation Family with Aniridia. Pedigree of 4 generation family that includes 19 individuals with aniridia. Seven members have a previously reported *PAX6* (NM_000280.4) c.565TC > T frame shift mutation that results in a premature stop codon. An additional seven individuals with aniridia were related by marriage in the second generation

Twelve patients were members of a previously described 4-generation family, in which genetic testing on generations iii, and iv showed a splice-site mutation (c.565TC > T) in *PAX6* (NM_000280.4) [27]. Seven additional patients were related to this family by marriage (Fig. 1).

Other familial relationships within our cohort included: father with 3 children, mother with 2 children, father and son, brother and sister twin siblings, and 2 sister pairs. In addition to the aforementioned splice-site mutation, 1 patient with familial aniridia had 11p14.1-p13 deletion (951 Kb loss) and 1 patient had 8p23.2 duplication of unknown significance found by chromosomal microarray analysis (Supplemental Table 1). Within the sporadic group, 4 patients had a confirmed mutation involving *PAX6* or the neighboring *ELP4* gene. Five patients with WAGR syndrome had gene deletions or duplications involving 11p14. Interestingly, 1 patient who clinically had WAGR syndrome had a frameshift mutation (found by exon-level targeted comparative genomic hybridization) which affected *PAX6*, but did not involve *ELP4*, *DCDC1*, or *WT1*. This patient presented at 4 months of age with bilateral partial aniridia with an iris stump, bilateral posterior polar cataracts, foveal hypoplasia, mild optic nerve hypoplasia, and roving-type nystagmus; he reacted equally to light in both eyes and IOP was 12 mmHg and 13 mmHg in the right and left eyes, respectively.

**Fig. 2:**
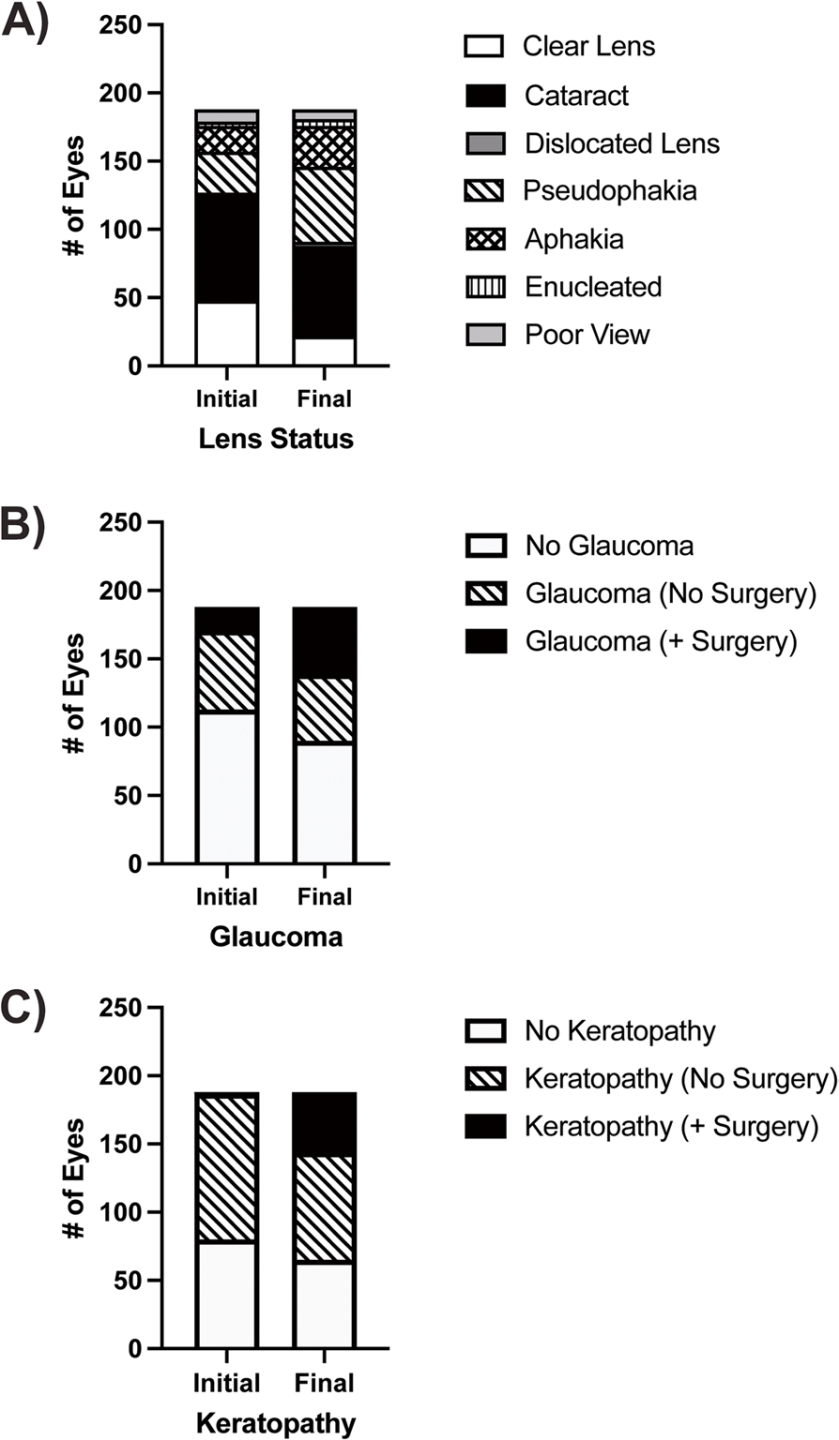
Proportion of Aniridic Individuals with Cataract, Glaucoma and Keratopathy. **A** At presentation, 66% of aniridic eyes were phakic while 26% had undergone cataract surgery and were either pseudophakic (16%) or aphakic (10%). The remaining eyes were either enucleated (2%) or the lens status was unknown (5%) due to corneal opacification. At final follow-up, 48% of the eyes were phakic, 29% were pseudophakic, 16% were aphakic, 3% were enucleated and 4% had unknown status due to corneal pacification. **B** Forty percent of eyes had a diagnosis of glaucoma at presentation and 9% had already undergone at least 1 IOP-lowering surgery. Glaucoma diagnosis increased to 52% at final follow-up and 26% of eyes required glaucoma surgery. **C** At presentation, 57% of aniridic eye had keratopathy and 0.5% had undergone corneal surgery. At final follow-up, the percent of eyes with keratopathy increased to 65% and 23% had at least 1 corneal surgery

At median follow-up of 4.0 years (mean 8.7 ± 12.0 years, range 0.1–53.8), 112 eyes (60%) of 62 patients (66%) underwent at least 1 surgery. Of these patients, 12 had unilateral surgery while 50 had bilateral surgeries. The average number of surgeries was 1.74 ± 2.30/eye (median 1, range 0–14). At presentation, the crystalline lens was present in 125 eyes (66%) of 66 patients (70%), of which 79 of 125 (63%) had cataract (Fig. 2A). Forty-nine eyes (26%) of 31 patients (33%) had previously undergone lensectomy, with 30 eyes (59%) having intraocular lens (IOL) implantation and 19 eyes (37%) remaining aphakic. Three eyes (2%) of 3 patients (3%) had enucleations prior to presentation and 9 eyes (5%) of 7 patients (7%) had unknown lens status due to corneal opacification. At final follow-up, 91 eyes (48%) of 52 patients (55%) remained phakic, although 61 eyes had a cataract and 3 eyes showed partial lens dislocation. Thus, an additional 36 eyes underwent lensectomy such that at final follow-up 85 eyes (45%) of 51 patients (54%) were pseudophakic and 30 eyes (16%) of 20 patients (21%) were aphakic. Only 1 eye had capsular tension ring (CTR) placement during lensectomy. Four eyes of 3 patients initially had IOL placement, which was later removed due to dislocation. Two additional eyes were enucleated during follow-up. Seven eyes (4%) of 6 patients (6%) had corneal opacification that precluded lens status at final follow-up.

At presentation, 75 eyes (40%) of 39 patients (41%) had glaucoma, of which 17 eyes (23%) of 12 patients (31%) had undergone prior IOP-lowering surgery (Fig. 2B). At final follow-up, 98 eyes (52%) of 50 patients (53%) had glaucoma, of which 49 eyes of 28 patients required IOP-lowering surgery with an average of 1.9 ± 1.6 surgeries/eye (range 1–10, median 1). Due to the extensive length of follow-up and conversion from paper to electronic records, accurate survival times were unable to be calculated for 54 of 94 glaucoma surgeries performed. Survival times of less than half of the surgeries would not yield accurate results, however, success rates at final follow-up were determined (Table 1). In the 35 eyes which underwent glaucoma drainage device placement (GDD), the average age at time of surgery was 28.2 ± 22.6 years (median 33.0 years, range 0.6–58.2 years).Success at final follow-up of Baerveldt and Ahmed GDDs were 74% and 63%, respectively. This increased to 87% and 88% if failure criteria did not include tube revision. Success of trabeculectomy with anti-fibrotics at final follow-up was 24%, which increased to 33% if bleb revision surgeries were excluded. Angle surgery showed 33% success at final follow-up. Success of cycloablation at final follow-up was 33% which increased to 50% if multiple cycloablations were excluded. There was no significant difference in length of follow-up between glaucoma surgeries (*p* > 0.2).

**Table 1:** Glaucoma Surgery

Glaucoma Surgery (n)	Previous Glaucoma Surgeries			Success at Final Follow-up	Success Exclusion	Success with Exclusion at Final Follow-up	Length of Follow-up (yrs)
	0 surgeries	1 surgery	> 2 surgeries				
**Glaucoma Drainage Device (35)**	23 eyes	7 eyes	5 eyes	71% (25 of 35 Eyes)	Tube Revision	89% (31 of 35 Eyes)	14.2 ± 15.4
** Baerveldt (23)**	16 eyes	4 eyes	3 eyes	74% (17 of 23 Eyes)		87% (20 of 23 Eyes)	13.2 ± 14.3
** Ahmed (8)**	3 eyes	3 eyes	2 eyes	63% (5 of 8 Eyes)		88% (7 of 8 Eyes)	17.5 ± 20.1
** Unspecified (4)**	4 eyes			75% (3 of 4 Eyes)		100% (4 of 4 Eyes)	2.7 ± 3.6
**Trabeculectomy with Anti-Fibrotics (21)**	14 eyes	6 eyes	1 eye	24% (5 of 21 Eyes)	Bleb Revision	33% (7 of 14 Eyes)	21.7 ± 14.1
**Angle Surgery (9)**	8 eyes	1 eye		33% (3 of 9 Eyes)	Repeat Angle Surgery	33% (3 of 9 Eyes)	16.3 ± 17.8
** Trabeculotomy (5)**	4 eyes	1 eye		40% (2 of 5 Eyes)		40% (2 of 5 Eyes)	26.6 ± 15.3
** Goniotomy (4)**	4 eyes			25% (1 of 4 Eyes)		25% (1 of 4 Eyes)	0.9 ± 1.3
**Cycloablation (12)**	3 eyes	4 eyes	5 eyes	33% (4 of 12 Eyes)	Multiple Cyclo-Ablations	50% (6 of 12 Eyes)	23.4 ± 12.0
** Contact Transcleral (5)**	1 eye	1 eye	3 eyes	40% (2 of 5 Eyes)		60% (3 of 5 Eyes)	19.8 ± 7.4
** Endoscopic (6)**	2 eyes	3 eyes	1 eye	17% (1 of 6 Eyes)		33% (2 of 6 Eyes)	20.5 ± 8.6
** Cryo (1)**			1 eye	100% (1 of 1 Eye)		100% (1 of 1 Eye)	43.4

**Fig. 3:**
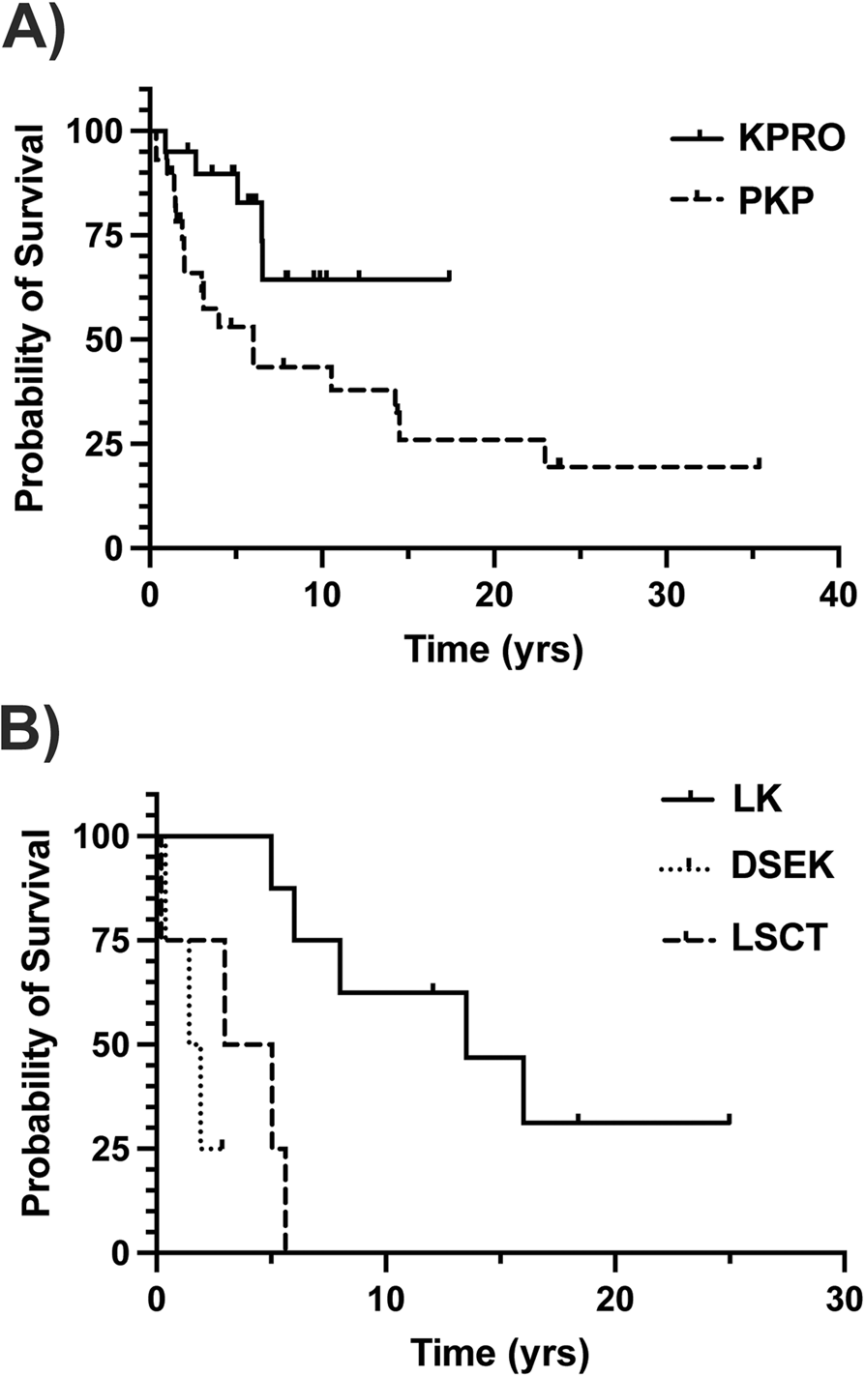
Survival Curves of Corneal Surgery in Aniridia. **A** Kaplan–Meier survival rates of penetrating keratoplasty (PKP) were 90% with 95% CI [71, 96] at 1-year, 43% with 95% CI [23, 62] at 10-years, and 20% with 95% CI [6,40] at 25-years. Keratoprosthesis (KPro) survival rate at 1-year, 5-years, and 10-years was 95% with 95% CI [70, 99] and 83% with 95% CI [55, 94] at 5-years, and 64% with 95% CI [32, 84], respectively*.* **B** Survival rates of limbal stem cell transplantation (LSCT) were 75% with 95% CI [13, 96] at 1-year and 25% with 95% CI [1, 66] at 5-years. Descemet stripping and endothelial keratoplasty (DSEK) 1-year survival rate was 75% with 95% CI [13, 96] and decreased to 25% with 95% CI [1, 66] at 2 years. Five-year, 10-year, and 15-year survival rates of lamellar keratoplasty (LK) were 88% with 95% CI [40, 99], 63% with 95% CI [23, 87], and 31% with 95% CI [6, 64], respectively

At presentation, 108 eyes (57%) of 56 patients (60%) had keratopathy, including 1 eye which had already undergone Boston keratoprosthesis (KPro) implantation (Fig. 2C). At final follow-up, 123 eyes (65%) of 63 patients (67%) had documented keratopathy, of which 44 eyes (36%) of 28 patients (44%) underwent at least 1 corneal procedure. The average number of corneal surgeries was 1.7 ± 1.1/eye (range 1–6, median 1). Corneal surgeries included penetrating keratoplasty (PKP, 19 eyes), Kpro (18 eyes), limbal stem cell transplantation (LSCT, 12 eyes), lamellar keratoplasty (LK, 4 eyes), and Descemet stripping endothelial keratoplasty (DSEK, 3 eyes). Kaplan–Meier analysis showed that PKP (Fig. 3A) had 1, 10, and 25-year survival rates of 90% with 95% CI [71, 96], 43% with 95% CI [23, 62], and 20% with 95% CI [6, 40], respectively. Of the 18 eyes which underwent KPro, 11 had not undergone prior corneal surgery while 6 had a history of failed PKP and 1 had previous LK. KPro survival rates were 95% with 95% CI [70, 99] at 1-year, 83% with 95% CI [55, 94] at 5-years, and 64% with 95% CI [32, 84] at 10-years. Of the 18 eyes which underwent KPro implantation, 7 eyes of 7 patients had repeat KPro surgery after the initial implant had failed. KPro failures included exposure secondary to blunt trauma (1 eye of 1 patient), dehiscence/exposure without a history of trauma (3 eyes of 3 patients), corneal melt with wound leaking (3 eyes of 2 patients), and neovascular membrane formation resulting in a tractional retinal detachment (1 eye of 1 patient). DSEK (Fig. 3B) 1-year survival rate was 75% with 95% CI [13, 96] and decreased to 25% with 95% CI [1, 66] at 2 years. LK had 5-year, 10-year, and 15-year survival rates of 88% with 95% CI [40, 99], 63% with 95% CI [23, 87], and 31% with 95% CI [6, 64], respectively. LSCT showed a 1-year survival rate of 75% with 95% CI [13, 96] and 5-year survival rate of 25% with 95% CI [1, 66]. Log-rank (Mantel Cox) analysis showed a significant difference between survival curves of the aforementioned corneal surgeries (*p* = 0.0006).

Additional surgeries included pars plana vitrectomy (PPV) in 20 eyes (11%) and artificial iris implantation in 4 eyes (2%). None of the iris implants resulted in any complication or required explantation. In 13 eyes, PPV was performed in conjunction with GDD implantation (7 eyes) and/or KPro placement (6 eyes). Two additional eyes had PPV combined with trabeculectomy or PKP. Five eyes underwent PPV to repair retinal detachments that were due to prior surgery (3 eyes), trauma (1 eye) or an optic nerve pit (1 eye). One eye, in addition to PPV, also had placement of scleral buckle for a KPro-related retinal detachment.

Nystagmus was present in 71 patients and absent in 3 patients. However, nystagmus status was not documented in 20 patients. Foveal hypoplasia was recorded in 46 patients (96 eyes) and optic nerve abnormalities (pallor, hypoplasia, dysplasia, pit) were reported in 13 patients. Foveal and optic nerve anatomy were not described in 29 patients due to inadequate view to the posterior segment.

**Fig. 4:**
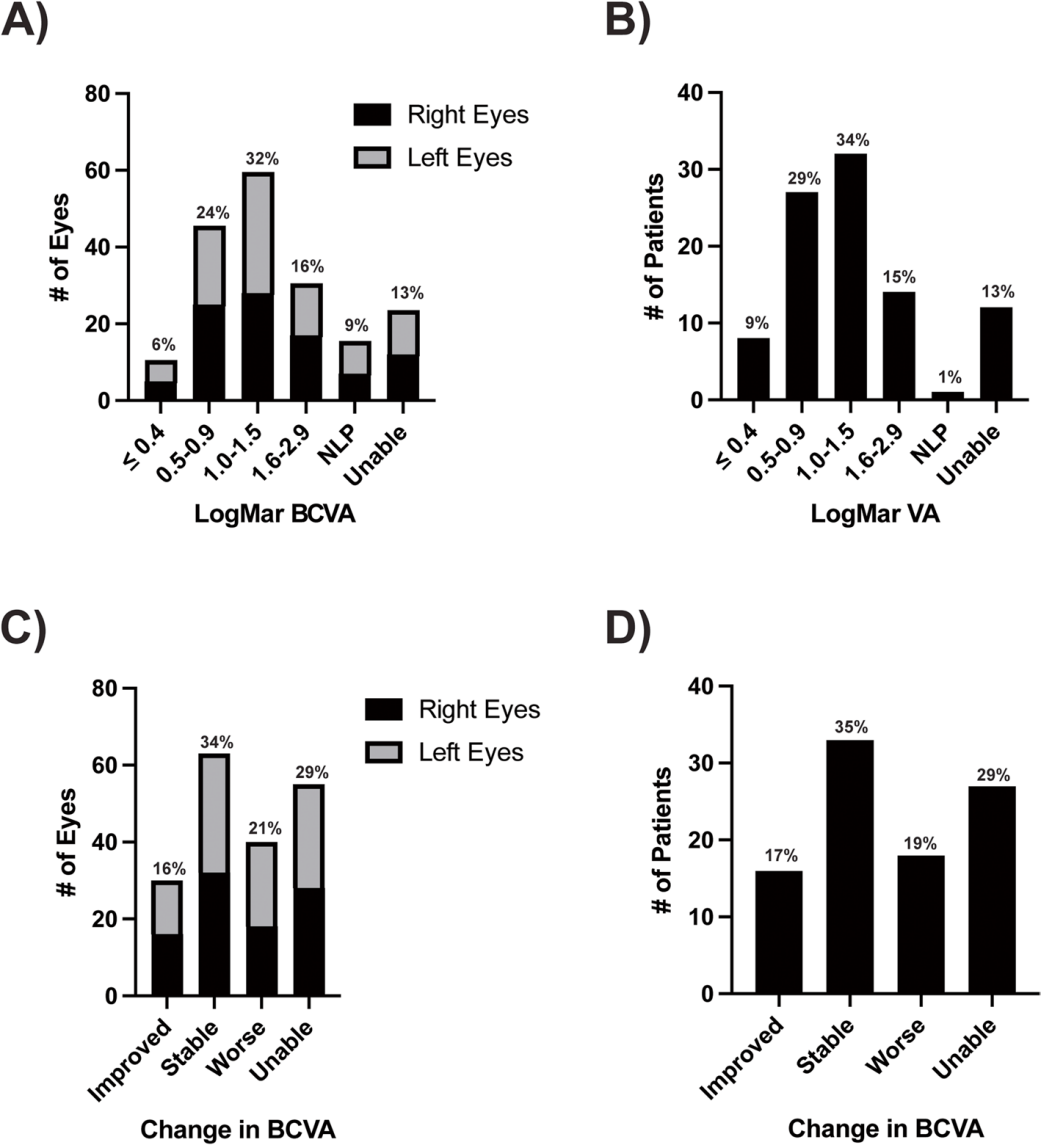
Visual Acuity Outcomes. **A** At final follow-up of the 188 eyes, 30% of eyes had LogMAR VA ≤ 0.9 (BCVA better than 20/200) and 6% had LogMAR VA ≤ 0.4 (BCVA 20/50 or better). Fifty-seven percent of eyes had LogMAR VA ≥ 1.0 (BCVA 20/200 or worse) with 9% unable to detect light. Thirteen percent of patients were too young to cooperate with optotype testing. **B** Analysis of the better seeing eye showed that 50% of the patients met the legal definition of blindness with LogMAR VA ≥ 1.0 (20/200 or worse) at final follow-up. One percent of patients were not able to detect light with either eye. Thirty-eight percent of patients had LogMAR VA ≤ 0.9 (better than 20/200) and 9% of patients had LogMAR VA ≤ 0.4 (20/50 or better). **C** In the 133 of 188 eyes that were able to complete optotype visual acuity testing at both initial and final visits, 34% of eyes showed stable VA (< 0.1 change in LogMAR VA) and 16% of eyes had an improvement in VA. Twenty-one percent of eyes showed worse LogMAR VA at final follow-up. Twenty-nine percent of patients lacked optotypte testing at either presentation or final follow-up. **D** Analysis of the better seeing eye showed that 35% of patients had stable VA (< 0.1 change in LogMAR VA) at initial and final follow-up. Seventeen percent of patients had improved LogMAR VA while 19% had worse VA at final follow-up compared to presentation

LogMAR BCVA at presentation (1.46 ± 0.46, median 1.0) was not significantly different (*p* = 0.51) than at final follow-up (1.32 ± 0.76, median 1.10). At final follow-up, 57 eyes (30%) had BCVA better than 20/200 with 11 (6%) having BCVA of 20/50 or better (Fig. 4A). In contrast, 107 eyes (57%) had BCVA worse than 20/200 with 16 eyes (9%) with no light perception. Twenty-four eyes (13%) were too young to cooperate with optotype testing at final follow-up. Analysis of the better seeing eye in each patient (Fig. 4B) showed similar results with 38% with BCVA better than 20/200. However, 50% met the legal definition of blindness (LogMAR ≥ 1.0) in the better seeing eye at final follow-up. Optotype visual acuity testing was completed at both initial and final visits in 133 eyes (71%) of 67 patients (71%). Ninety-three eyes (50%) had stable (< 0.1 change in LogMar VA) or improved BCVA (Fig. 4C) while 40 eyes (21%) showed worse BCVA at final follow-up. Likewise, BCVA of the better seeing eye (Fig. 4D) improved or remained stable in 52% of patients, but decreased in 19% of patients.

Univariate analysis of all 188 eyes showed that final BCVA was associated with BCVA at initial presentation (*p* < 0.001), age at initial presentation (*p* < 0.0001), glaucoma diagnosis (*p* < 0.001), cataract at presentation (*p* = 0.037), and presence of keratopathy at presentation (*p* < 0.0001). While there was no difference in final BCVA between phakic and pseudophakic eyes, aphakic eyes showed worse BCVA compared to pseudophakic (*p* < 0.005) and phakic (*p* < 0.005) eyes. Furthermore, eyes which underwent PKP or KPro implantation were associated with worse BCVA (*p* < 0.0001 and *p* < 0.05, respectively). There was no relationship between final BCVA and LSCT, glaucoma surgery, retina surgery, or iris prosthetic implantation. Univariate and multivariate analyses both showed that neither sex nor race were associated with final BCVA.

## Discussion

Congenital aniridia involves almost all ocular structures, and therefore affects vision via numerous mechanisms [1, 2]. This study presents the clinical courses and visual outcomes in the largest US-based cohort of aniridic patients to date and aims to improve prognostication and management of this disease.

Cataracts are common in aniridia with rates of 40–80% [1, 18–20]. Lens extraction in aniridia presents unique challenges including altered anterior chamber dynamics, abnormal capsule and zonule properties, and corneal opacification [14, 28]. This coupled with the decreased viability of the sulcus as an alternative location for an IOL, makes lensectomy complicated. There are few studies on outcomes of cataract surgery in aniridia [14, 28]. One Chinese study with 17 eyes of 10 patients showed improvement in vision and decrease in photophobia after cataract surgery with CTR and IOL placement [14]. In our study, 68% of individuals at presentation either were noted to have a cataract or had already undergone lens extraction and this increased to 80% at final follow-up. The presence of cataract at presentation was associated with worse BCVA at final follow-up. This is in contrast to a Korean study in which cataract at initial or final visit was not associated with visual outcome [1]. However, Chang et al. focused on the pediatric population, with 85% of their cohort being younger than 3 years. Furthermore, there were fewer patients (60), a lower rate of cataract formation (53%), and ultimately only 25% of the eyes underwent lensectomy. In our study, 85 eyes (45%) underwent cataract surgery, and the 30 eyes which were aphakic had worse outcomes compared to pseudophakic and phakic eyes. Factors such as amblyopia and contact lens intolerance, may compromise visual function in aphakic individuals. Furthermore, the surgical decision to leave an eye aphakic may have been due to severe corneal scarring or complicated intraoperative findings, which may also result in decreased visual acuity.

Glaucoma affects at least 50% of individuals with aniridia [3, 29, 30]. The mechanisms underlying increased IOP include iridogoniodysgenesis and iris stump anterior rotation leading to angle closure. We found that glaucoma was associated with worse visual outcomes. Further, Jain et al. showed that higher baseline IOP and multiple glaucoma surgeries were associated with worse visual outcomes [31]. Angle surgery, and namely goniotomy, has been advocated as a prophylactic measure to lower the iris root to decrease its anterior rotation [29, 30]. Since this would need to be done prior to glaucoma onset and has the risk of lens and cornea damage, this has largely fallen out of favor. In aniridic eyes with elevated IOP, angle surgery has a success rate that ranges from 0–80% with trabeculotomy more successful than goniotomy. However, these reports are hampered by limited follow-up and few patients [29, 30, 32]. In the current study, angle surgery was not performed prophylactically, but only in eyes with elevated IOP. At final follow-up, one-third of eyes which underwent angle surgery remained successful, which is a much lower rate compared to other childhood glaucomas [33, 34]. Thus, while trabeculotomy and goniotomy can be performed in aniridic glaucoma, it behooves the surgeon to prepare the patient for subsequent IOP-lowering surgeries.

Trabeculectomy also has limited success in aniridia. In Wiggins and Tomey, trabeculectomy in 17 eyes had a success rate of 9% [9]. More recently, an Indian study showed 42% success rate of trabeculectomy with mitomycin C at 2-years in 12 eyes [35]. In our study, we found a 33% success rate in 21 eyes. Because of low success and life-long infection risk, GDDs have supplanted trabeculectomy for many surgeons. In 3 small cohorts, Ahmed GDDs showed a 67–87% survival rate at 1-year, and Baerveldts had 88% success with less than 3 years of follow-up [12, 15, 36]. In our study, Baerveldt devices in 23 eyes showed 74% success while Ahmeds in 8 eyes had 63% success at final follow-up which ranged between 0.5–22 years. These studies suggest that GDDs are more successful than trabeculectomy and angle surgery in obtaining IOP-control in aniridia. Nonetheless, it is important to note specific considerations with GDDs in aniridia. Intraoperative placement is difficult due to shallow anterior chambers and minimal lens protection. Furthermore, the combination of iris hypoplasia and limbal stem cell deficiency dictates careful placement of the tube such that it minimizes contact with both the lens and corneal endothelium [4, 25, 35, 36].

Keratopathy is present in up to 80% of individuals with aniridia [4, 37, 38]. Early scarring may be treated medically or by superficial keratectomy with amniotic membrane grafting. LSCT is an option in moderate keratopathy. Prior studies show a greater than 70% success of LSCT with improvement in vision [25]. However, allogenic and not autologous stem cells must be transplanted, which typically necessitates systemic immunosuppression [25]. In the current study, LSCT showed poor success with complete failure in all eyes after 5 years. This may be due to keratopathy severity and follow-up length. Of note, none of the 12 LSCT performed were performed combined with corneal transplantations. With severe scarring, corneal transplantation is typically the next step. PKP and partial thickness grafts both show high failure rates in aniridia due to limbal stem cell deficiency [4, 25, 39]. In our cohort, DSEK had 25% survival at 2-years, while LK and PKP had similarly low survival rates after 15-years such that many eventually ended up with a KPros.

While previous studies have shown modest success of KPros implants, there are long-term complications such as glaucoma, retinal detachment, extrusion, endophthalmitis, and phthisis [11, 13, 16, 26, 40, 41]. In our cohort, KPros showed the highest rates of survival at 5 and 10-years compared to other corneal surgeries, but given this relatively new technology, length of follow-up in these eyes was shorter than other surgeries. Furthermore, recent studies have shown that KPro implantation after PKP graft failure results in better outcomes compared to repeat PKP [40]. It is important to note that our study showed that both PKP and KPro implantation were associated with worse BCVA at final follow-up. Although both procedures are prone to failure in aniridia, it is likely that the corneal opacification, which was the original indication was already severely limiting vision. Thus, realistic expectations regarding potential complications and likelihood of additional surgeries must be set with aniridic patients undergoing corneal transplantation or keratoprosthesis surgery.

The strengths of our study are the large number of eyes and patients and the long length of follow-up. Limitations include the retrospective nature of the case series, variable time of follow-up, and variable disease severity at initial presentation. In addition, the intraocular surgeries (cataract, glaucoma, and cornea) were performed by multiple surgeons who had different criteria for intervention and various surgical techniques. There also may have been changes in surgical techniques over the duration of follow-up making comparison between surgical success difficult. Furthermore, given the retrospective nature of this study, there was no consistency in genetic evaluation; most patients had undergone targeted sequencing of PAX6, especially patients who underwent initial evaluation several decades ago. It is possible that mutations in other genes associated with anterior segment dysgeneses may have been missed, although this is becoming less common with recent advancements in whole exome sequencing [42].

In conclusion, congenital aniridia is a complex disease with multiple mechanisms that underlie both early and progressive vision loss. Treatment is life long and requires a good understanding of the risks unique to aniridia. In this large US-based cohort of patients with congenital aniridia, we found that most patients underwent at least 1 eye surgery and that cataract, glaucoma, and keratopathy were associated with worse BCVA at final follow-up. This study helps determine important prognostic factors and guide treatments that will yield the best visual outcomes in congenital aniridia.

## Conclusions

In this large US-based cohort, the majority of patients with congenital aniridia underwent at least 1 intraocular surgery. At least half of patients developed glaucoma and keratopathy. Cataract, glaucoma, and keratopathy were associated with worse visual acuity.

## Supplementary Information

**Additional file 1: Supplemental Table 1. **Genetics.

## Data Availability

The datasets used and/or analyzed during the current study are available from the corresponding author (BLB).

## Abbreviations

VA: Visual Acuity
BCVA: Best Corrected Visual Acuity
IOP: Intraocular Pressure
