# Supplementary material for: Clinical outcomes and visual prognostic factors in congenital aniridia
Source: BMC Ophthalmol. 2022 May 25;22:235. doi: 10.1186/s12886-022-02460-5 (PMC9131660; doi:10.1186/s12886-022-02460-5)
Supplement: Supplementary file 1 — Additional file 1: Supplemental Table 1. Genetics. [file 12886_2022_2460_MOESM1_ESM.docx]

**Supplemental Table 1: Genetics**

| Genetic Mutation | NM Number | Mutation Mechanism | Aniridia Type |
| --- | --- | --- | --- |
| *PAX6*  c.565TC>T  p.Ile190Ser fsTer17 | NM_000280.4 | Frameshift mutation resulting in premature stop codon | Familial - 7 individuals (see Figure 1) |
| *PAX6*  del (11p14.1-p13) |  | 951 Kb deletion involving *PAX6* | Familial - 1 individual |
| dup (8p23.2) |  | Unknown significance | Familial - 1 individual |
| *ELP* exon  del (11p13) |  | Deletion involving *ELP* Exons 6-10 | Sporadic |
| *PAX6*  c.183C>G  p.Tyr75* | NM_001604.5 | Nonsense mutation in *PAX6* resulting in premature stop codon | Sporadic |
| *PAX6*  c.949C>T  p.Arg317Ter | NM_000280.4 | Nonsense mutation on *PAX6* resulting in premature stop codon | Sporadic |
| *PAX6* exon*, ELP* exon  del (11p13) |  | Deletion involving *ELP4* Exon 10 and *PAX6* Exons 8-13 | Sporadic |
| *PAX6, WT1*  del (11p14.1-p12) |  | 10.6 Mb deletion involving 52 genes including *WT1* and *PAX6* | WAGR |
| *PAX6, WT1*  del (111p14.1-11p13)  dup (11q14.2) x2 |  | 6.5 Mb deletion involving ET1 and PAX6  2 separate duplications (387 Kb and 160 Kb) at 11q14.2 of unknown significance | WAGR |
| *PAX6, WT1*  del (11p13) |  | 1.8 Mb deletion involving *WT1* and *PAX6* | WAGR |
| *PAX6, WT1, BDNF*  del (11p13-p11.2) |  | 13.1 Mb deletion involving *WT1*, *PAX6* and *BDNF* | WAGR |
| *PAX6, WT1*  del (11p14.1-p12) |  | 9.18 Mb deletion involving 51 genes including *WT1* and *PAX6* | WAGR |
| *PAX6*  c.502delG  p.Val168Cys fsX39 | NM_000280.4 | Frameshift mutation resulting in premature stop codon | WAGR |
